# The aetiology of breast cancer subtypes: results from the Million Women Study

**DOI:** 10.1186/s13058-025-02197-1

**Published:** 2025-12-28

**Authors:** Gillian Reeves, Kirstin Pirie, Sarah Floud, Judith Black, Krystyna Baker, Toral Gathani

**Affiliations:** Cancer Epidemiology Unit, Nuffield Department of Population Health, University of Oxford, Richard Doll Building, Roosevelt Drive, Oxford, OX3 7LF UK

## Abstract

**Background:**

Evidence regarding the aetiology of specific breast cancer subtypes may provide insights into the mechanisms underlying their development, and improve prevention of rarer but more aggressive subtypes. We investigated risk factor associations with surrogate molecular subtypes of breast cancer in a large cohort of UK women.

**Methods:**

In 1.2 million postmenopausal women aged 50–64 recruited into the Million Women Study in 1996–2001, we estimated risks of breast cancer subtypes (defined by oestrogen receptor [ER], progesterone receptor [PR], and human epidermal growth factor receptor 2 [HER2] status) in relation to established risk factors for breast cancer.

**Results:**

Among 1,228,671 eligible women, followed on average for 19.8 (SD 6.5) years, there were 58,134 incident breast cancers with known ER status and 40,627 with known surrogate molecular subtype (based on ER, PR, and HER2 status). Most established risk factors were primarily either positively (age at first birth, age at menopause, BMI, height, alcohol intake, and menopausal hormone therapy use) or inversely (parity) associated with ER+ cancer (p-value for heterogeneity by ER status < = 0.002 in each case). Only prior oral contraceptive (OC) use showed a greater association with ER than with ER+ cancer (*p* = 0.002). Some additional differences were observed by surrogate molecular subtype including a modest positive association of parity, and inverse association of breastfeeding, with the risk of basal-like cancer.

**Conclusions:**

Most established risk factors for breast cancer are almost exclusively associated with hormone-sensitive cancers but some have definite associations with ER- cancers (prior OC use), or more specifically, with basal-like cancer (parity, breastfeeding).

**Supplementary Information:**

The online version contains supplementary material available at 10.1186/s13058-025-02197-1.

## Introduction

Breast cancer is known to be a heterogeneous disease and early genomic studies have identified a number of molecular subtypes including luminal A, luminal B, HER2-enriched, and basal-like cancers [1], which are closely related to key immunohistochemical markers such as oestrogen receptor (ER), progesterone receptor (PR), and human epidermal growth factor 2 (HER2) status. Although these molecular subtypes have been associated with differences in clinical outcomes [2], their aetiological relevance remains unclear.

Studies of the aetiology of breast cancer subtypes can provide insights into the biological mechanisms underlying their development and may also provide a means of identifying women at greatest risk of particular subtypes, who may benefit from targeted prevention or screening interventions. In particular, there is increasing interest in models which predict the risk of basal-like and HER2-enriched cancers, which are less likely to be detected at screening, and have a relatively poor prognosis [3]. 

The vast majority (>80%) of breast cancers diagnosed each year among UK women aged 50 or above are luminal hormone-sensitive cancers [4], and so associations of factors with overall breast cancer risk in this population largely reflect their relationship with such cancers. For this reason, reliable evidence regarding risk factors for rarer, more aggressive, subtypes including HER2-enriched and basal-like cancers can only be obtained from extremely large studies of the general population, or from relatively large studies of populations with a greater risk of such cancers (for example, younger women or those of African ancestry). Although gene-expression analysis of tumour tissue is not feasible on a very large scale, immunohistochemistry markers (ER, PR, and HER2 status) can be used to derive broad surrogate molecular subtypes for the purposes of epidemiological studies [5]. 

We report here on associations between established risk factors and breast cancer subtypes defined by ER, PR and HER2 status, in a large prospective study of 1.2 million postmenopausal UK women.

## Methods

### Data collection and definitions

In 1996–2001, 1.3 million women aged 50–64 were recruited into the Million Women Study (MWS) through the NHS breast screening programme. At recruitment, participants provided information about sociodemographic factors, reproductive history, and other personal characteristics, and have since been re-surveyed at 3–5 year intervals (full details are given elsewhere [6] and at http://www.millionwomenstudy.org). Women have been followed up for cancers and deaths via linkage to routinely collected healthcare records. All study participants gave written informed consent to take part in the study. Ethical approval for the MWS was provided by the Oxford and Anglia Multi-Centre Research Ethics Committee (MREC ref: 9/57/001).

### Classification of tumours by molecular subtype

Cancers were classified according to the International Classification of Diseases, 10th Revision (ICD-10), with invasive breast cancer defined as C50. Information on ER, PR and HER2 status was primarily obtained from routinely collected cancer registration data but where such information was missing, relevant information from breast cancer audit data, medical records or questionnaire data was used, where available. Cancers were grouped by ER status, by PR status within ER+ cancers, and by combinations of ER, PR, and HER2 status [(ER+/PR+, HER2-), (ER+/PR+, HER2+), (ER-, PR-, HER2+), (ER-, PR-, HER2-)], which were taken to represent the four main molecular subtypes (luminal A, luminal B, HER2-enriched, and basal-like cancers). In sensitivity analyses aimed at assessing the impact of alternative classifications, luminal cancers were further differentiated on the basis of grade with luminal A cancers defined as ER+/PR+, HER2-, grade 1/2 and luminal B cancers defined as ER+/PR+, HER2-, grade 3 or ER+/PR+, HER2+.

### Statistical analysis

Cox regression models were used to obtain estimated hazard ratios (henceforth referred to as relative risks) for each of the breast cancer subtypes considered. Women were excluded if they had a previous registration of invasive cancer (excluding non-melanoma skin cancer, ICD-10 code C44), or of in situ breast cancer (ICD-10 code D05). Women contributed person-years from recruitment to the earliest of: registration with any cancer (except non-melanoma skin cancer C44), death, or end of follow-up (31st December 2022). Women who were not known to be postmenopausal at recruitment were entered into analyses from the first survey at which they reported being postmenopausal or their 55th birthday, whichever was earliest.

Analyses were routinely stratified by geographical region (10 cancer registry areas in the UK) and adjusted for age at recruitment and quintiles of area-based deprivation index [7], and were mutually adjusted, as appropriate, for height, body mass index (BMI), smoking status, frequency of strenuous exercise, alcohol consumption, age at menarche, parity, age at first birth, duration of oral contraceptive (OC) use, age at menopause, menopausal hormone therapy (MHT) use, and first-degree family history of breast cancer. MHT use was included as a time dependent variable, and set to unknown from 4 years after a woman last reported information on her use. Time since last OC use was also treated as a time dependent variable. Women with missing values for any adjustment variable were assigned to a separate category for that variable. Because the effects of BMI and age at menopause on breast cancer risk are altered in users of MHT[8], analyses of these factors were restricted to never MHT users.

In plots comparing risks across more than two categories, variances were estimated using the floating absolute risks approach [9] and results presented in the form of relative risks (RRs) and “group-specific” confidence intervals (g-s CIs). In the text, standard 95% CIs are given.

The main analyses were based on molecular subtypes defined by ER, PR and HER2 status only, but sensitivity analyses were also conducted in which luminal A and B cancers were defined using tumour grade in addition to ER, PR and HER2 status. There is likely to be some degree of misclassification of ER and other immunohistochemical markers [10], although assay performance is likely to have improved over time. Therefore, where a risk factor was found to have a significant but much lesser association with ER- than ER+ cancer, consideration was given to whether the observed association with ER- cancer could plausibly be due to misclassification of ER status. In addition, sensitivity analyses were conducted in which follow-up was restricted to the period after 2010. All analyses were done using Stata (version.18.5).

## Results

Analyses included 1,228,671 women aged 55 (IQR 52–60) years on average at baseline. During a mean follow-up period of 19.8 (SD 6.5) years, there were 58,134 incident breast cancers with known ER status, of which 31,844 (55%) had information on PR status, and 41,867 (72%) had information on HER2 status. Table 1 summarises the distribution of included cases by ER, PR and HER2 status, and the average age at diagnosis for each subtype. The average age at diagnosis was broadly similar across all cancer subtypes. This is likely to reflect improvements in completeness of registry information on ER, PR and HER2 over time, with cancer registrations after 2010 being considerably more likely to have information on all three markers than those diagnosed prior to 2010. The vast majority of cancers (87%) were ER+, and among cancers with known surrogate molecular subtype (based on ER, PR and HER2 status), 81%, 9%, 3%, and 7%, were classified as luminal A, luminal B, HER2-enriched, and basal-like, respectively.

**Table 1:** Distribution of breast cancer cases by ER, PR and HER2 status, among those with known ER status

Tumour characteristic	Number of cancers	Mean (SD) age at diagnosis
*ER status*		
ER-	7676 (13%)	68.7 (7.7)
ER+	50,458 (87%)	68.8 (7.3)
*PR status*		
PR-	9095 (29%)	70.2 (7.2)
PR+	22,749 (71%)	70.0 (7.0)
Not known	*26,290*	
*HER2 status*		
HER2-	36,639 (88%)	70.7 (6.4)
HER2+	5228 (12%)	70.1 (6.8)
Not known	*16,267*	
*ER, PR status (ER+ cancers)*		
ER+, PR-	4289 (16%)	70.0 (7.2)
ER+, PR+	22,455 (84%)	70.0 (7.0)
Not known	*23,714*	
*ER, PR and HER2 status*		
ER+ or PR+, HER2- *	32,865 (81%)	70.7 (6.3)
ER+ or PR+, HER2+	3703 (9%)	70.0 (6.7)
ER-, PR-, HER2	1143 (3%)	70.5 (7.0)
ER-, PR-, HER2	2916 (7%)	71.2 (6.7)
Not known	*17,507*	

*26,873 grade 1 or 2, 5518 grade 3

**Fig. 1:**
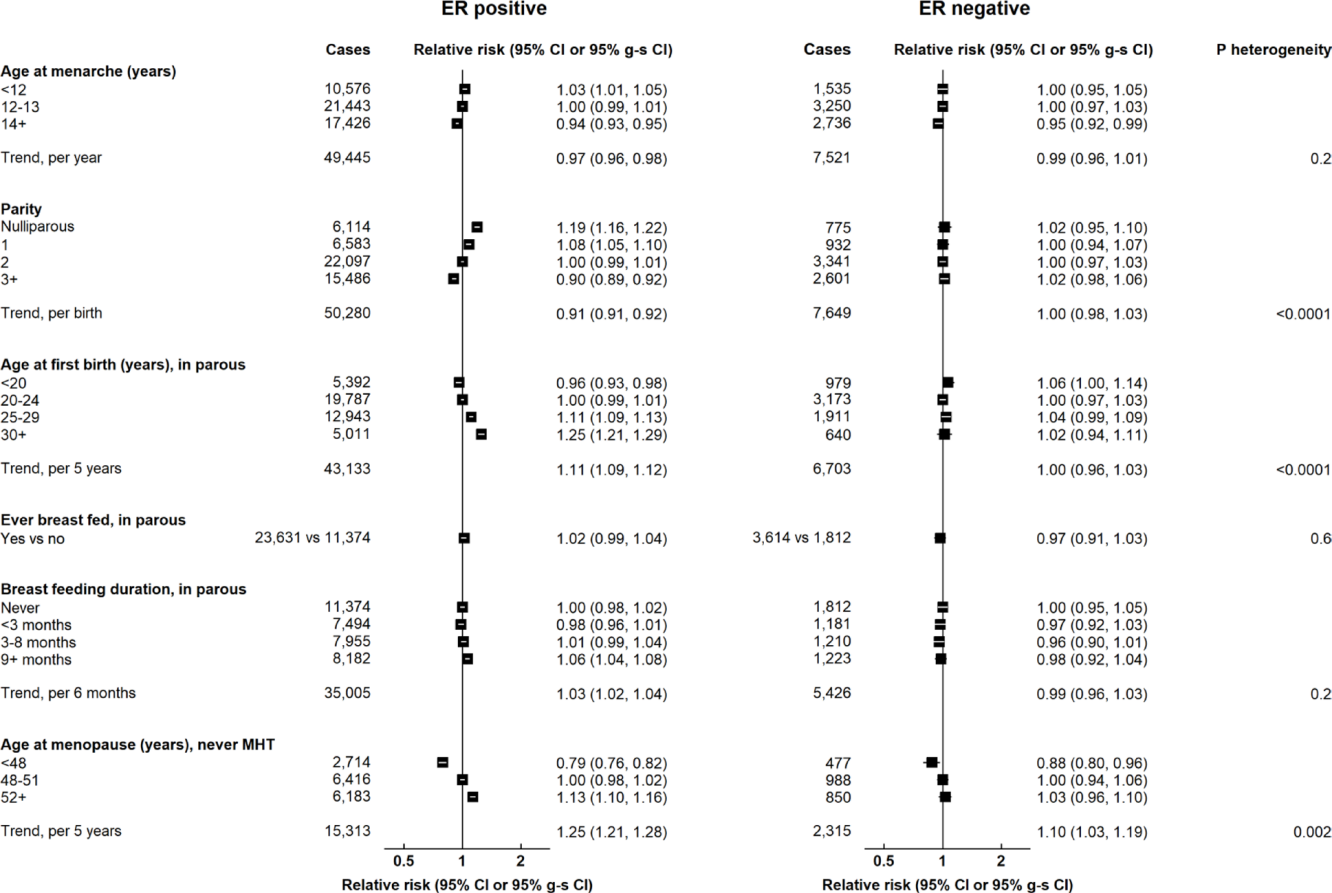
Associations of reproductive factors with breast cancer risk by ER status. Tests for heterogeneity are by cancer subtype. g-s CI = group-specific confidence interval

Almost all reproductive factors had substantial associations with ER+ breast cancer (Fig. 1). Earlier menarche and later menopause were associated with a higher risk of ER+ cancer (RR per 1 year earlier age at menarche = 1.03, 1.02–1.04; RR per 5 year later age at menopause = 1.25, 1.21–1.28); higher parity was associated with a lower risk of ER+ disease (RR per birth = 0.91, 0.91–0.92); and later age at first birth was associated with a higher risk (RR per 5 year increase in age first birth = 1.11, 1.09–1.12). Most of these factors had a much lesser, or no, association with ER- disease [p-value for heterogeneity by ER status: <0.0001 (age at first birth, parity); *p* = 0.002 (age at menopause)]. There was little association of ever breastfeeding or duration of breastfeeding with ER+ cancer (RR per 6 months duration = 1.03, 1.02–1.04) or ER- cancer (RR = 0.99, 0.96–1.03).

**Fig. 2:**
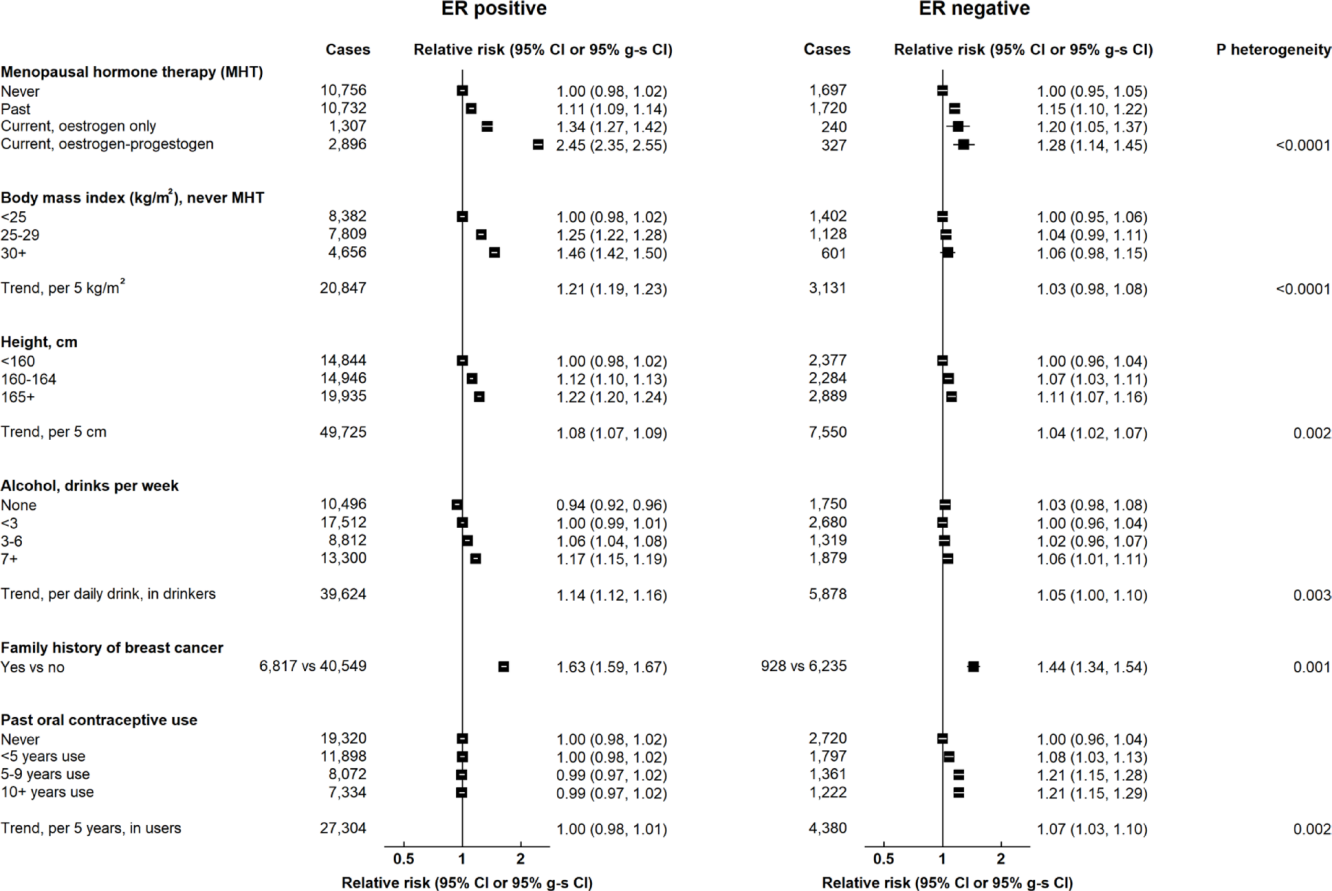
Associations of non-reproductive factors with breast cancer risk by ER status. Tests for heterogeneity are by cancer subtype. g-s CI = group-specific confidence interval

Three other risk factors showed substantial associations with ER+ cancer and much lesser, or null, associations with ER- cancer (Fig. 2). Current use of combined and oestrogen-only MHT was associated with RRs of 2.45 (2.34–2.57) and 1.34 (1.26–1.43), respectively, for ER+ cancer, and 1.28 (1.13–1.46) and 1.20 (1.03–1.39), for ER- cancer (p-value for heterogeneity < 0.0001); for BMI, the relative risk per 5 unit increment was 1.21 (1.19–1.23) for ER+ cancer and 1.03 (0.98–1.08) for ER- cancer (*p* < 0.0001); and for alcohol intake, the RR per additional daily drink in drinkers was 1.14 (1.12–1.16) for ER+ cancer and 1.05 (1.00–1.10) for ER- cancer (*p* = 0.003). There was less difference between associations of height with ER+ (RR per 5 cm increment = 1.08, 1.07–1.09) and ER- cancer (1.04, 1.02–1.07) (p-value for heterogeneity = 0.002) and between associations of family history of breast cancer with ER+ (RR = 1.63, 1.59–1.67) and ER- cancer (1.44, 1.34–1.54) (p-value for heterogeneity = 0.001). In contrast, duration of past OC use was associated with ER- disease (RR per 5 years past use = 1.07, 1.03–1.10) but not ER+ disease (RR = 1.00, 0.98–1.01) (p-value for heterogeneity = 0.002).

In analyses by ER and PR status, associations of age at menarche and BMI with ER+ disease were largely confined to ER+ PR+ cancers (p-values for heterogeneity by PR status = 0.07 and < 0.0001, respectively), and the association of combined MHT use with ER+ cancers was notably greater for ER+ PR+ than for ER+ PR- cancers (p-value for heterogeneity = 0.0003) (eFigures 1 and 2).

**Fig. 3:**
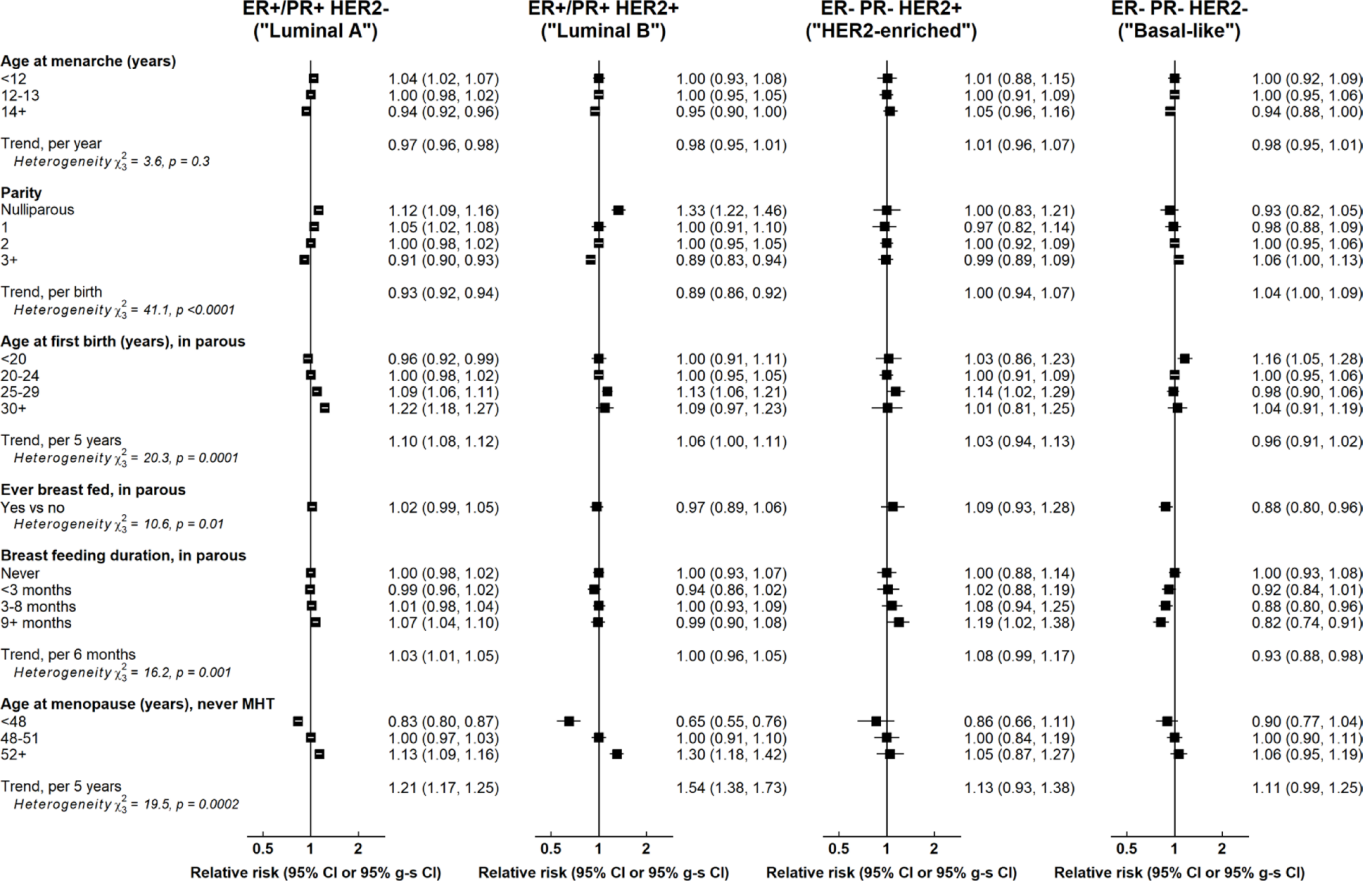
Associations of reproductive factors with breast cancer risk by molecular subtype. Tests for heterogeneity are by cancer subtype. g-s CI = group-specific confidence interval

There was significant variation in associations of all reproductive factors, except age at menarche, by molecular subtype (Fig. 3). This was generally largely accounted for by differences between luminal versus non-luminal subtypes, but in the case of parity and breastfeeding there appeared to be additional qualitative differences by finer molecular subtypes. In particular, parity was positively associated with basal-like cancers (RR per birth = 1.04, 1.00–1.09) and breastfeeding showed a distinct inverse association with basal-like cancers in parous women (RR for ever breastfeeding = 0.88, 0.80–0.96; RR per 6 months duration of breastfeeding = 0.93, 0.88–0.98), which appeared to be evident at each level of parity (eFigure 3). There were also a number of statistically significant but relatively minor quantitative differences in associations by molecular subtype including a slightly greater inverse association of parity with luminal B (RR per birth = 0.89, 0.86–0.92) than with luminal A (RR = 0.93, 0.92–0.94) cancers (p-value for heterogeneity = 0.007), and a slightly greater association of age at menopause with luminal B than with luminal A cancers (RR per 5 year increase: 1.54, 1.38–1.73 vs. 1.21, 1.17–1.25; p-value for heterogeneity < 0.0001).

**Fig. 4:**
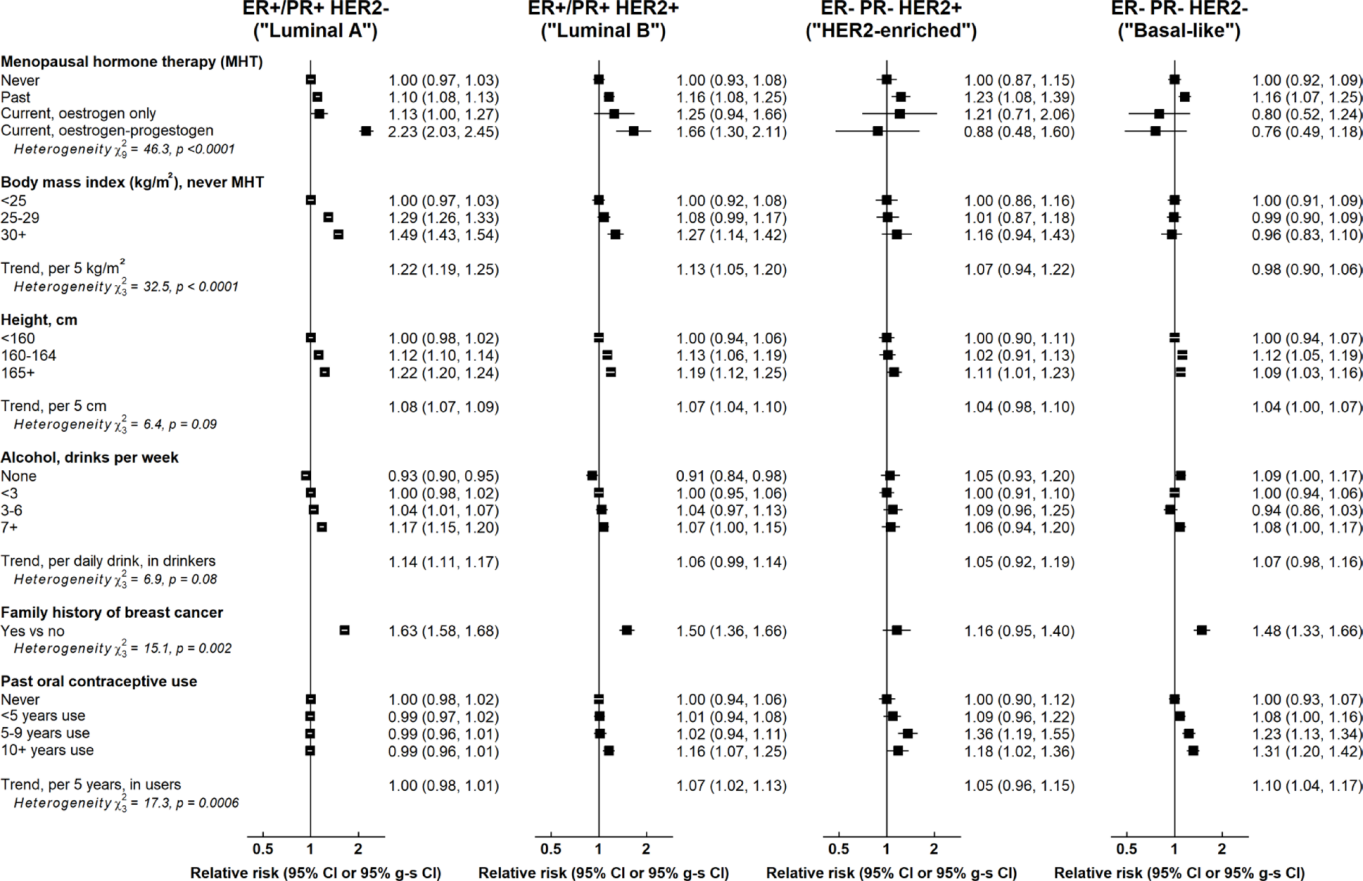
Associations of non-reproductive factors with breast cancer risk by molecular subtype. Tests for heterogeneity are by cancer subtype. g-s CI = group-specific confidence interval

Differences in associations of non-reproductive factors by molecular subtype also mainly reflected differences in luminal versus non-luminal cancers (Fig. 4), with a few exceptions. Combined MHT use, BMI, and alcohol intake showed slightly greater associations with luminal A than luminal B cancers (p-values for heterogeneity = 0.03, 0.02, and 0.08, respectively). Family history had broadly similar positive associations with all subtypes, although there was some evidence of a lesser association with HER2-enriched cancers [RRs: luminal A (1.63, 1.58–1.68), luminal B (1.50, 1.36–1.66), basal-like (1.48, 1.33–1.66), HER2-enriched (1.16, 0.95–1.40); p-value for heterogeneity = 0.002]. Furthermore, duration of past OC use appeared to have a positive association with all subtypes apart from luminal A cancers [RRs per 5 years past use: luminal A (1.00, 0.98–1.01), luminal B (1.07, 1.02–1.13), HER2-enriched (1.05, 0.96–1.15), basal-like (1.10, 1.04–1.17); p-value for heterogeneity = 0.0006].

Sensitivity analyses in which the classification of luminal cancers was based on grade as well as ER, PR and HER2 status (eFigures 4–5), and in which follow-up was restricted to the period 2010 or later (eFigures 6–9), yielded broadly similar results.

## Discussion

In this large prospective study of 1.2 million women and over 50,000 breast cancers, we found substantial, and in some cases qualitative, differences in the effects of established risk factors for breast cancer by tumour subtype. The majority of risk factors considered, including those relating to childbirth, age at menarche/menopause, adiposity, height, alcohol intake and MHT use, appeared to be predominantly associated with ER+ or ER+ PR+ cancers. In contrast, family history and past OC use had definite associations with ER- cancers. Parity and breastfeeding were somewhat unusual in that they showed qualitatively different associations with basal-like cancers as compared with other subtypes. While some of these differences have been previously noted, a recent scoping review [11] concluded that, among White women at least, the only risk factor for which there was convincing evidence of heterogeneity in associations by ER+ and ER- subtypes was parity. Our findings therefore add considerably to our knowledge of the aetiology of specific subtypes among this group of women, providing novel and definitive evidence, in particular, for a qualitatively different effect of alcohol, OC use and breastfeeding on certain ER+ and ER- subtypes.

### Reproductive factors

Childbearing is known to have a greater effect on hormone sensitive than on other breast cancers [12] but there is limited data comparing its effects on specific subtypes. Two systematic reviews [13, 14] and a pooled re-analysis of prospective studies [15] found variation in associations of parity and/or age at first birth by molecular subtype which were largely driven by differences by ER+ status, but also in some cases by a distinct positive association of parity with basal-like cancers [13, 15]. In addition, two previous studies have examined the joint association of breastfeeding and parity on breast cancer subtypes [16, 17], one of which found a significantly lesser adverse association of parity with basal-like cancers in Black women who breastfed [17]. Our findings confirm the substantial protective effect of childbearing on luminal, hormone-sensitive cancers but also provide new evidence of a modest positive association of parity with basal-like cancers.

A large collaborative reanalysis reliably demonstrated that breastfeeding is associated with a reduction in overall risk of breast cancer [18]. Three systematic reviews and/or meta-analyses have since reported that breastfeeding is associated with a reduced risk of ER- PR-[19] and/or triple-negative cancers [13, 14, 19] but its association with ER+ subtypes remains unclear, with most evidence for an inverse association with luminal cancers or ER+ PR+ cancers coming from retrospective studies [19]. The findings presented here provide important new evidence that in the long-term at least, breastfeeding is not associated with ER+ subtypes but may mitigate an adverse effect of parity on basal-like cancer.

It is not entirely clear as to why childbearing might reduce the risk of ER+ cancers but increase the risk of triple-negative/basal-like cancers in the long-term. This differential pattern of risk is, however, consistent with previous findings indicating that although childbirth leads to an immediate increase in risk of both ER+ and ER- cancers, the increased risk of ER+ cancer subsequently declines, resulting in a decreased risk by around 25 years, but the increased risk of ER- cancer appears to persist [20]. Basal-like cancers are thought to arise from undifferentiated luminal progenitor cells [21], and the apparent mitigation of the increased risk of ER- cancer following childbirth afforded by breastfeeding may reflect the promotion of progenitor cell maturation caused by lactation [22]. Alternatively, it has been suggested that long-term breastfeeding may reduce the risk of breast cancer because it results in a more gradual and controlled involution process [23]. It has been hypothesised that the long-term protection of childbearing against ER+ cancer is due to high levels of sex hormones during pregnancy bringing about terminal differentiation of the breast epithelium, leading to reduced responsiveness to oestrogens and/or progesterone [24]. 

### Age at menarche and menopause

Pooled reanalyses of epidemiological studies have demonstrated that age at menopause, but not necessarily age at menarche, has a greater association with ER+ than ER- cancers [25], and that later age at menopause is associated with a greater increase in risk of luminal subtypes than with other molecular subtypes [15], although some systematic reviews have failed to find evidence of variation in associations by molecular subtype [13, 14]. Our findings confirm the greater effect of delayed menopause on ER+ subtypes, but also show that the effects of age at menarche are largely confined to ER+ PR+ cancers which represent a subset of ER+ cancers that are particularly hormone-sensitive [26]. These findings therefore provide further support for the idea that earlier menarche and later menopause increase risk through prolonging a woman’s exposure to endogenous sex hormones.

### Anthropometric factors

The positive association of adiposity with postmenopausal breast cancers, particularly ER+/PR+ cancers [27, 28], is well established, and a pooled analysis of prospective data has also shown that adiposity is predominantly associated with luminal molecular subtypes [15]. This is likely due to the fact that, after the menopause, greater adiposity leads to increased oestrogen synthesis and reduced circulating sex-hormone binding globulin, leading to higher levels of bioavailable oestradiol, which increases breast cancer risk [29]. Our finding that BMI-associated risk was confined to highly hormone-sensitive ER+ PR+ cancers supports this mechanism.

Height has previously been associated with a greater risk of ER+/PR+ than other breast cancers [28], which is in line with our findings. The mechanisms underlying this association are unclear but insulin like growth factors (IGFs) may have a role, as they are major regulators of growth during childhood and adolescence and IGF-I associated increases in ER+ breast cancer risk have been demonstrated in both observational and Mendelian randomization analyses [30]. Misclassification of ER status may have contributed to the modest association of height with ER- cancer since this effect was no longer evident for cancers diagnosed after 2010.

### Alcohol

Evidence regarding the effects of alcohol on breast cancer by ER status, or molecular subtype, is inconclusive. Pooled analyses of prospective studies have shown similar positive associations with ER+ and ER- cancer [31] and no significant variation in alcohol-associated risks by molecular subtype [15], but an earlier meta-analysis of case-control and prospective studies reported a slightly greater effect of alcohol in all ER+ versus ER- PR- cancers [32], and two cohort studies, not included in any of the above mentioned re-analyses, reported alcohol-associated risks that were largely confined to ER+ cancers [33, 34]. Our findings provide clear evidence that, in this group of middle-aged women, the adverse effects of moderate alcohol intake are largely confined to ER+ cancers, and are greatest for luminal A cancers.

Some intervention studies have shown that alcohol consumption is associated with acute increases in serum/plasma concentrations of oestrogens and/or androgens in pre- and/or post-menopausal women [35–39] although others found no significant effect [40–42]. Recent observational and Mendelian randomisation studies also found alcohol intake to be positively associated with higher serum levels of certain sex hormones, and lower levels of sex steroid hormone binding globulin, in pre- and postmenopausal women [43–45]. These findings, together with our observation that greater alcohol intake is predominantly associated with ER+ subtypes, suggest that alcohol acts mainly through increasing levels of bioavailable sex hormones, although it may also influence risk through other mechanisms, especially at higher intakes than were typical in this cohort.

### Exogenous hormone use

Use of oestrogen-only, and oestrogen-progestogen, MHT has been shown to have a greater positive association with ER+ breast cancers [8, 46]. Previous studies have, however, had limited power to examine MHT-associated risks by molecular subtype, and a pooled analysis of prospective studies found no evidence of differences in the association of ever MHT-use across the four main molecular subtypes [15]. Our findings confirm the substantial effect of current MHT use, particularly combined preparations, on risk of ER+ cancers but also provide clear evidence of a greater association of combined MHT use with luminal A than luminal B cancers, and little or no association with other molecular subtypes. The much smaller, albeit significant, association of recent MHT use with all ER- cancers observed here may be due, at least in part, to misclassification of ER status since there was no association with HER2-enriched or basal-like cancers, and information on recent MHT use in relation to all ER- cancers was almost exclusively from cases diagnosed before 2010, when ER measurement may have been less reliable.

Given the established relationship between endogenous hormones and hormone sensitive breast cancer, and the reduction in risk observed in users of anti-oestrogenic therapies such as tamoxifen [47], MHT is likely to increase risk through increasing circulating oestrogen levels. Why use of MHT containing a progestogen as well as an oestrogen should lead to a greater increase in risk than oestrogen-only MHT is less clear but is consistent with the higher breast epithelial cell proliferation seen in the luteal phase of the menstrual cycle when levels of both oestradiol and progesterone are high [48]. 

A large pooled re-analysis of 45 studies found convincing evidence that OC use is associated with a small transient increase in the overall risk of breast cancer [49] but did not report risks by breast cancer subtypes. A number of subsequent studies have examined associations of OC use with risk by ER/PR status [50–56] or by molecular subtype [13–15, 50, 56–58]. Some of these studies have demonstrated greater associations of OC use with risk of ER- than ER+ cancers[53, 55, 56], but there has been little clear evidence of variation in associations according to molecular subtype [13–15]. Given the age profile of our study, we were able to reliably investigate the long-term risks of OC use (i.e. 20 + years after stopping) and to demonstrate that prior OC use in this cohort of women is associated with an increased long-term risk of all molecular subtypes except luminal A cancer. The MWS did not collect information on the specific type of oral contraceptive used but the median year of stopping use was 1975 (IQR 1971–1979) and so they are likely to have predominantly used older generations of oral contraceptives containing potentially different progestins to those in use today. Further studies will be needed to investigate whether long duration use of other contemporary formulations show a similar persistent effect on risk.

It is unclear as to why OC use should increase the long-term risk of ER- but not ER+ disease, although there may be parallels with the differential effect of childbirth on risk by ER status [20]. Given the high prevalence of OC use, any effect on cancer risk which persists into older age is potentially of public health importance and further investigation is needed to ascertain whether this effect varies by type of OC, or by a woman’s personal characteristics.

### Family history

A family history of breast cancer has been shown to increase the risk of both ER+ and ER- breast cancer [59]. A systematic review [13], and a pooled analysis of prospective studies [15], both found that family history was positively associated with all four molecular subtypes, although the latter reported some variation in risk by molecular subtype, with the greatest associations observed with luminal B and triple-negative cancers. Our findings broadly concur with these findings in that we found family history to be associated with similar increases in the risk of ER+ and ER- cancers. However, our finding of a lower association of family history with HER2-enriched compared with all other molecular subtypes is novel and needs to be confirmed in other large studies. Self-reported family history of breast cancer is likely to reflect contributions from many inherited factors, each of which may impact on one or more subtypes, so it is perhaps not surprising that it is associated with an increased risk of most molecular subtypes. It is unclear why it might have less impact on HER2-enriched tumours but may suggest a lesser role of inherited factors in the development of such tumours.

### Strengths and limitations

The main strengths of this study were the availability of prospectively collected data on a wide range of risk factors, and the extremely large sample size, including more than 40,000 breast cancers with information on surrogate molecular subtype based on ER, PR and HER2 status, providing more power to detect modest differences in associations by subtype than is typically available in an individual study. The categorisation of cancers by surrogate molecular subtype used in the main analyses was based solely on ER, PR and HER2 status, in line with many other large epidemiological studies. This categorisation results in a somewhat greater proportion of luminal A relative to luminal B cancers than is typically observed based on alternative categorisations which include grade. However, sensitivity analyses based on the latter approach to categorisation yielded similar results. Although we were able to demonstrate clear associations of many risk factors with specific surrogate molecular subtypes, more data are needed to fully elucidate the role of certain risk factors in the development of HER2-enriched and basal-like cancers. Since the vast majority of MWS participants were of White European ancestry, and postmenopausal at recruitment, we were unable to assess risk factors for breast cancer subtypes in pre-menopausal women or in women of different ancestries.

## Conclusions

Most established risk factors for breast cancer are almost exclusively associated with hormone sensitive cancer subtypes. In contrast, family history, OC use, parity and breastfeeding appear to have definite associations with some or all ER- subtypes, which in some cases are qualitatively different from their associations with ER+ cancers.

## Supplementary Information

Below is the link to the electronic supplementary material.

Supplementary Material 1

## Data Availability

Data from the Million Women Study are available to bona fide researchers in accordance with the Million Women Study Data Access Policy (https://www.ceu.ox.ac.uk/research/the-million-women-study/data-access-and-sharing/data-access-policy). Further information is available from the corresponding author upon request.
