# Supplementary material for: The aetiology of breast cancer subtypes: results from the Million Women Study
Source: Breast Cancer Res. 2025 Dec 28;28:30. doi: 10.1186/s13058-025-02197-1 (PMC12857095; doi:10.1186/s13058-025-02197-1)
Supplement: Supplementary file 1 — Supplementary Material 1 [file 13058_2025_2197_MOESM1_ESM.docx]

**Supplementary material**

**eFigure 1: Associations of reproductive factors with ER positive breast cancer risk by PR status**


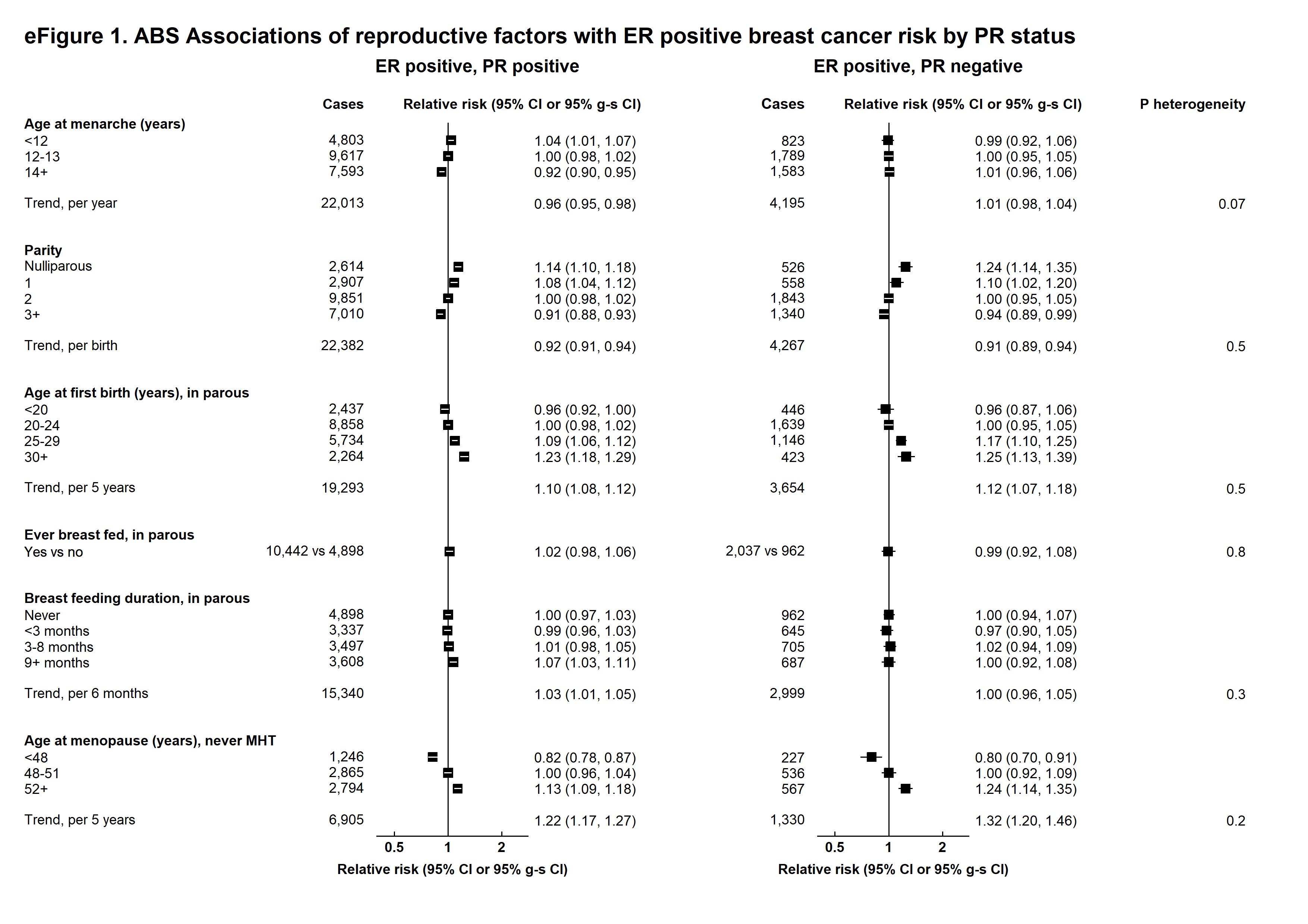


*Tests for heterogeneity are by cancer subtype. g-s CI = group-specific confidence interval*

**eFigure 2: Associations of non-reproductive factors with ER positive breast cancer risk by PR status**

**
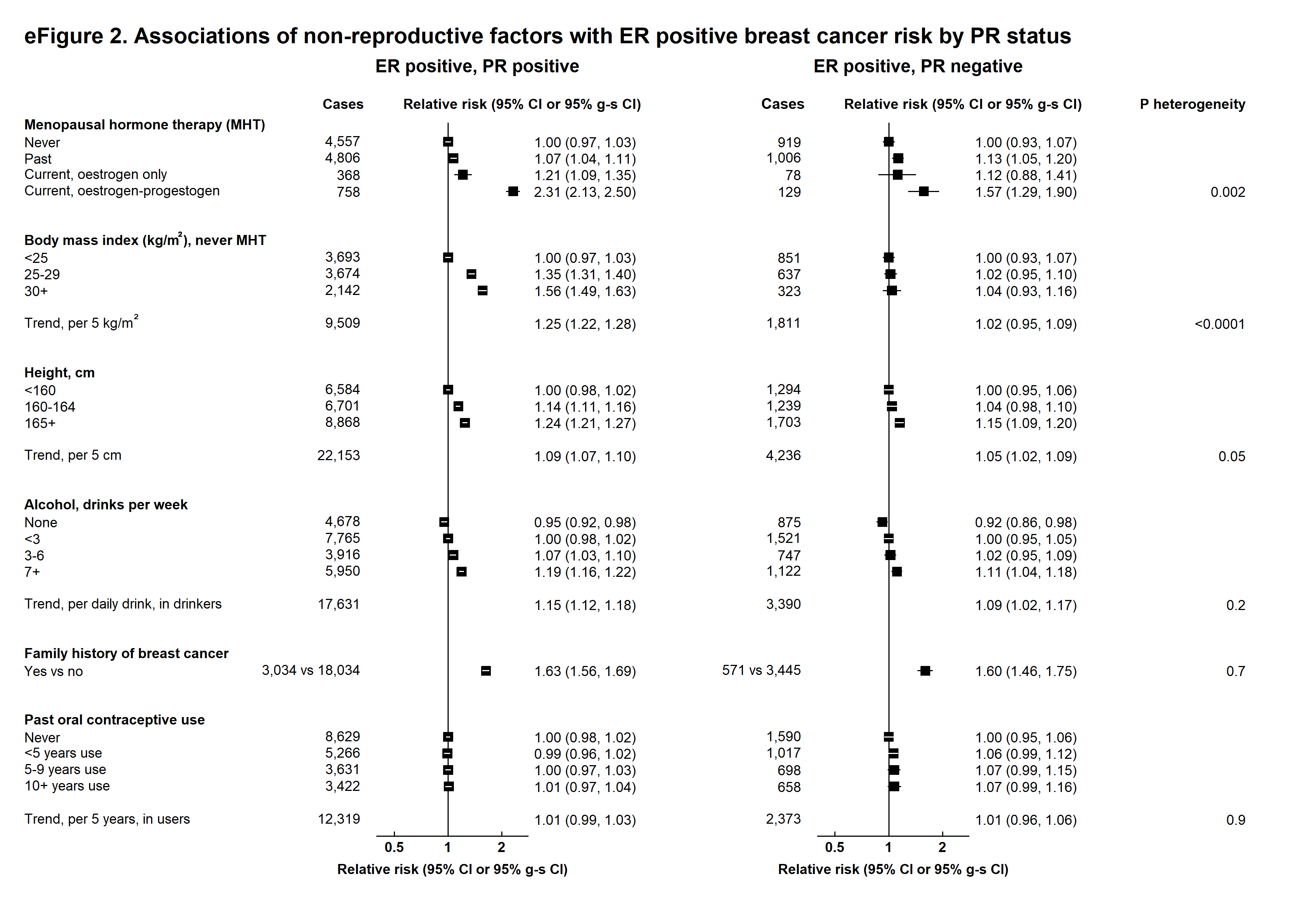
**

*Tests for heterogeneity are by cancer subtype. g-s CI = group-specific confidence interval*

**eFigure 3: Associations of parity and breast feeding with breast cancer risk by molecular subtype**

**
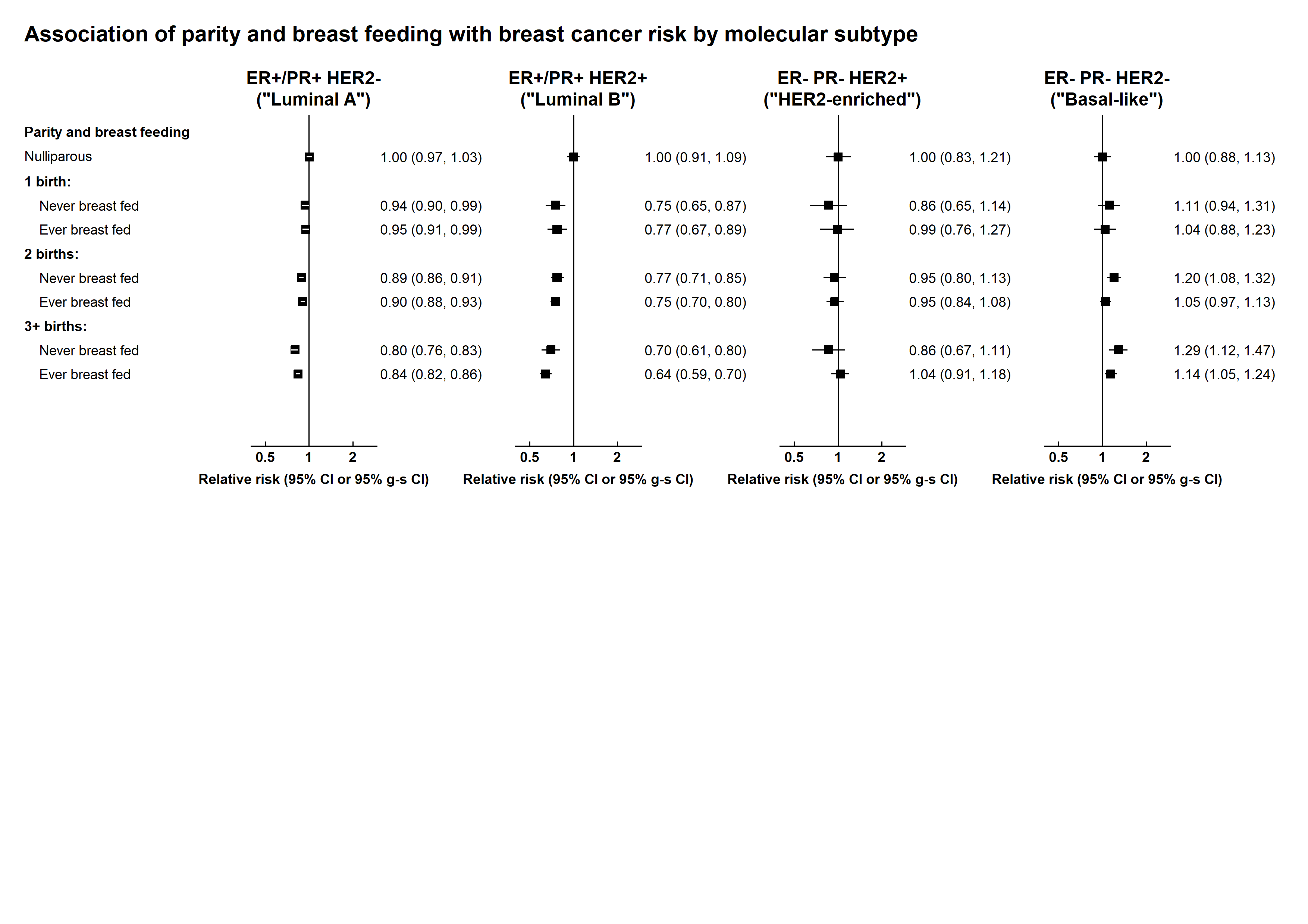
**

*Tests for heterogeneity are by cancer subtype.* *g-s CI = group-specific confidence interval*

**eFigure 4: Associations of reproductive factors with breast cancer risk by surrogate molecular subtype defined using ER status, PR status, HER2 status and grade**

**
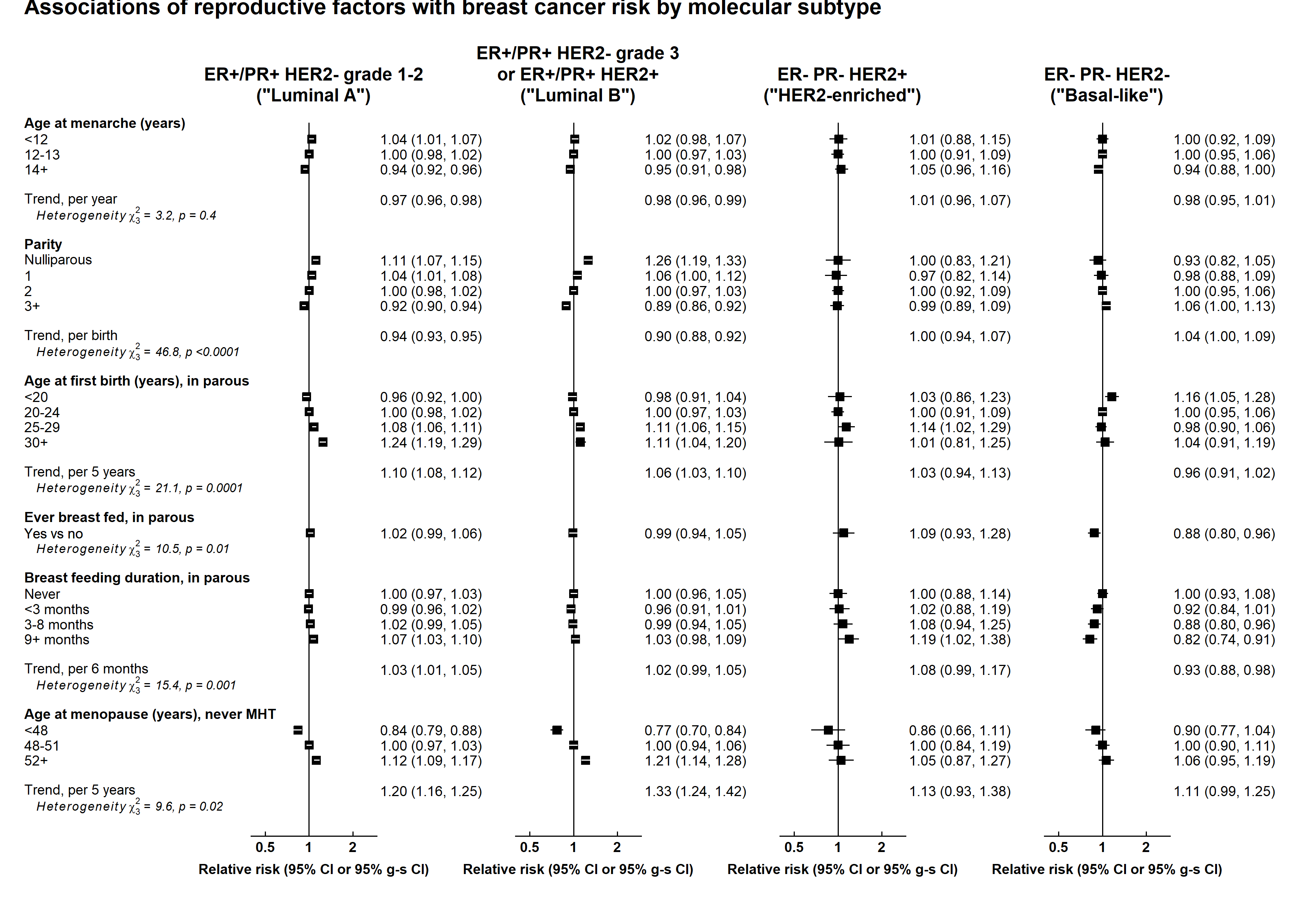
**

*Tests for heterogeneity are by cancer subtype. g-s CI = group-specific confidence interval*

**eFigure 5: Associations of non-reproductive factors with breast cancer risk by surrogate molecular subtype defined using ER status, PR status, HER2 status and grade**

**
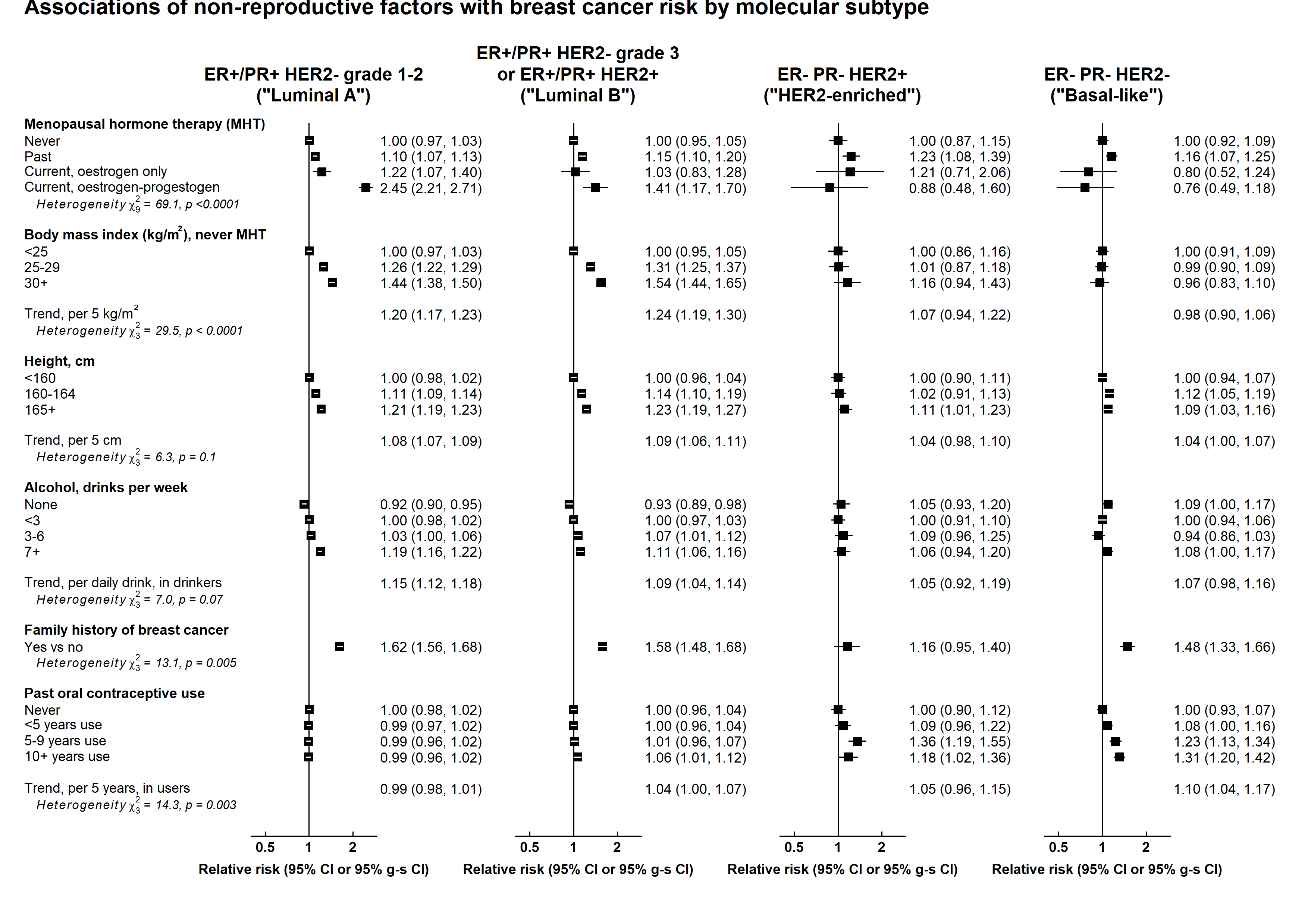
**

*Tests for heterogeneity are by cancer subtype. g-s CI = group-specific confidence interval*

**eFigure 6: Associations of reproductive factors with breast cancer risk by ER status, excluding breast cancers diagnosed prior to 1^st^ January 2010**


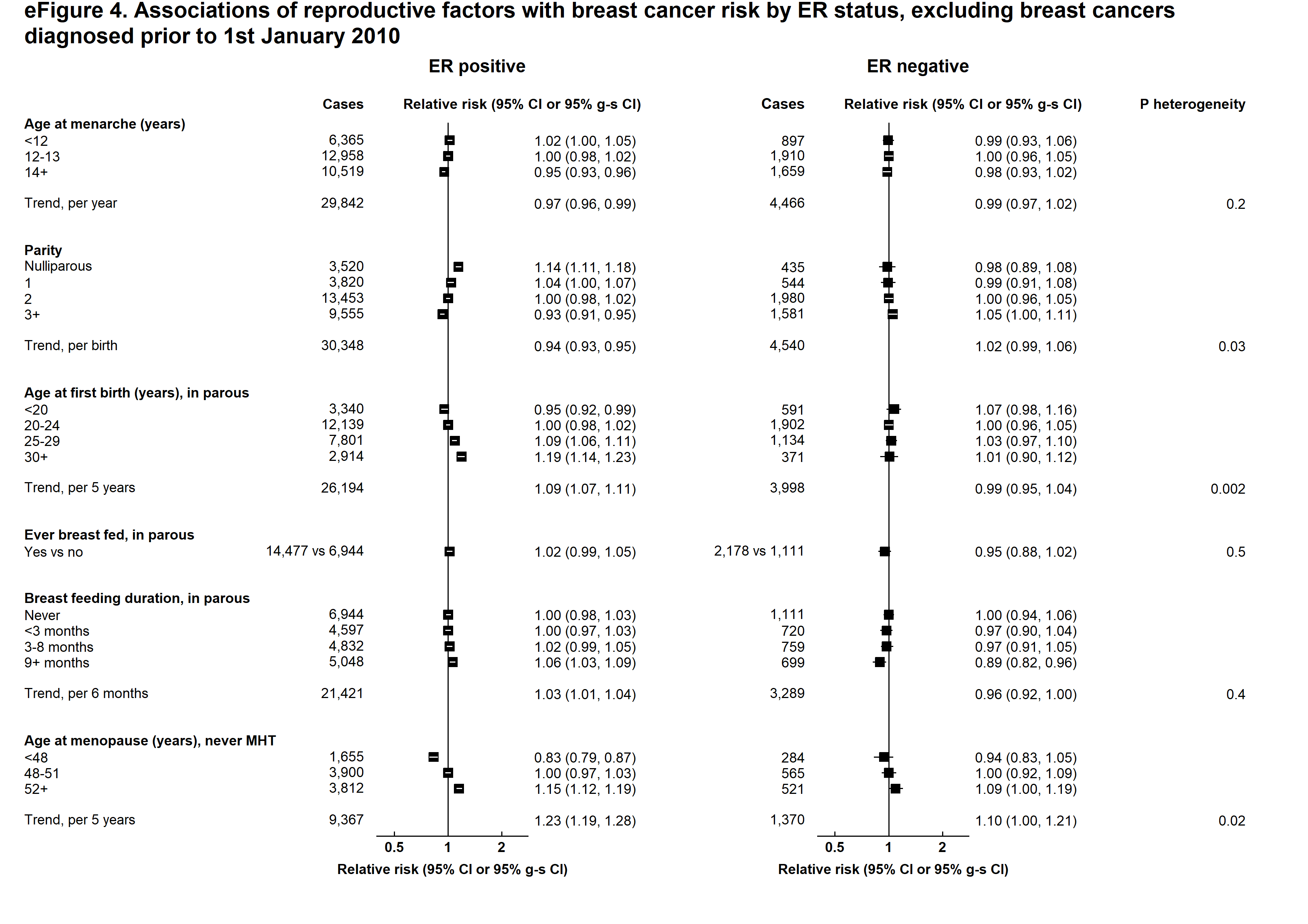


*Tests for heterogeneity are by cancer subtype. g-s CI = group-specific confidence interval*

**eFigure 7: Associations of non-reproductive factors with breast cancer risk by ER status, excluding breast cancers diagnosed prior to 1^st^ January 2010**

**
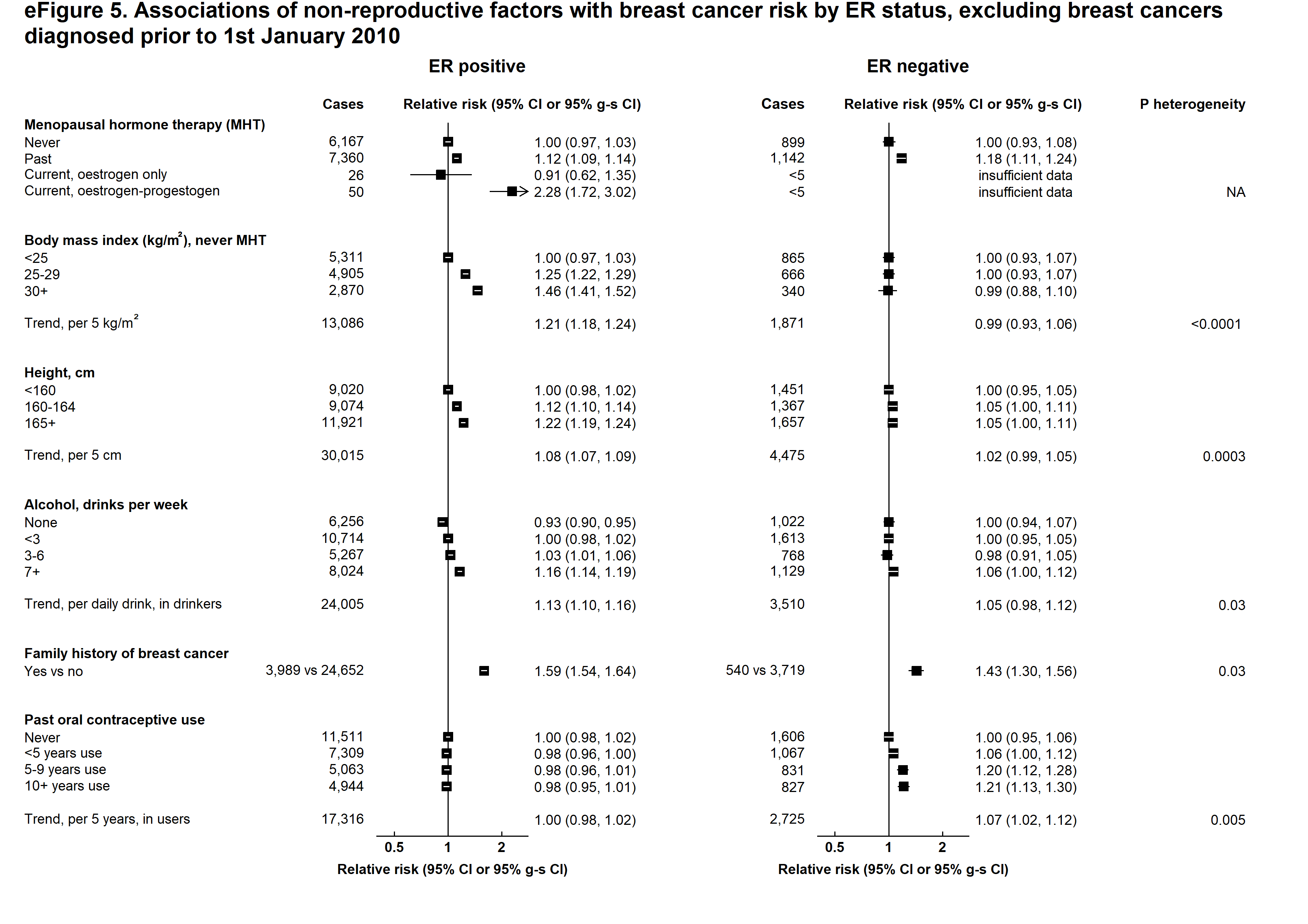
**

*Tests for heterogeneity are by cancer subtype. Results for groups with fewer than five events are marked as having “insufficient data”. g-s CI = group-specific confidence interval*

**eFigure 8: Associations of reproductive factors with breast cancer risk by surrogate molecular subtype, excluding breast cancers diagnosed prior to 1^st^ January 2010**


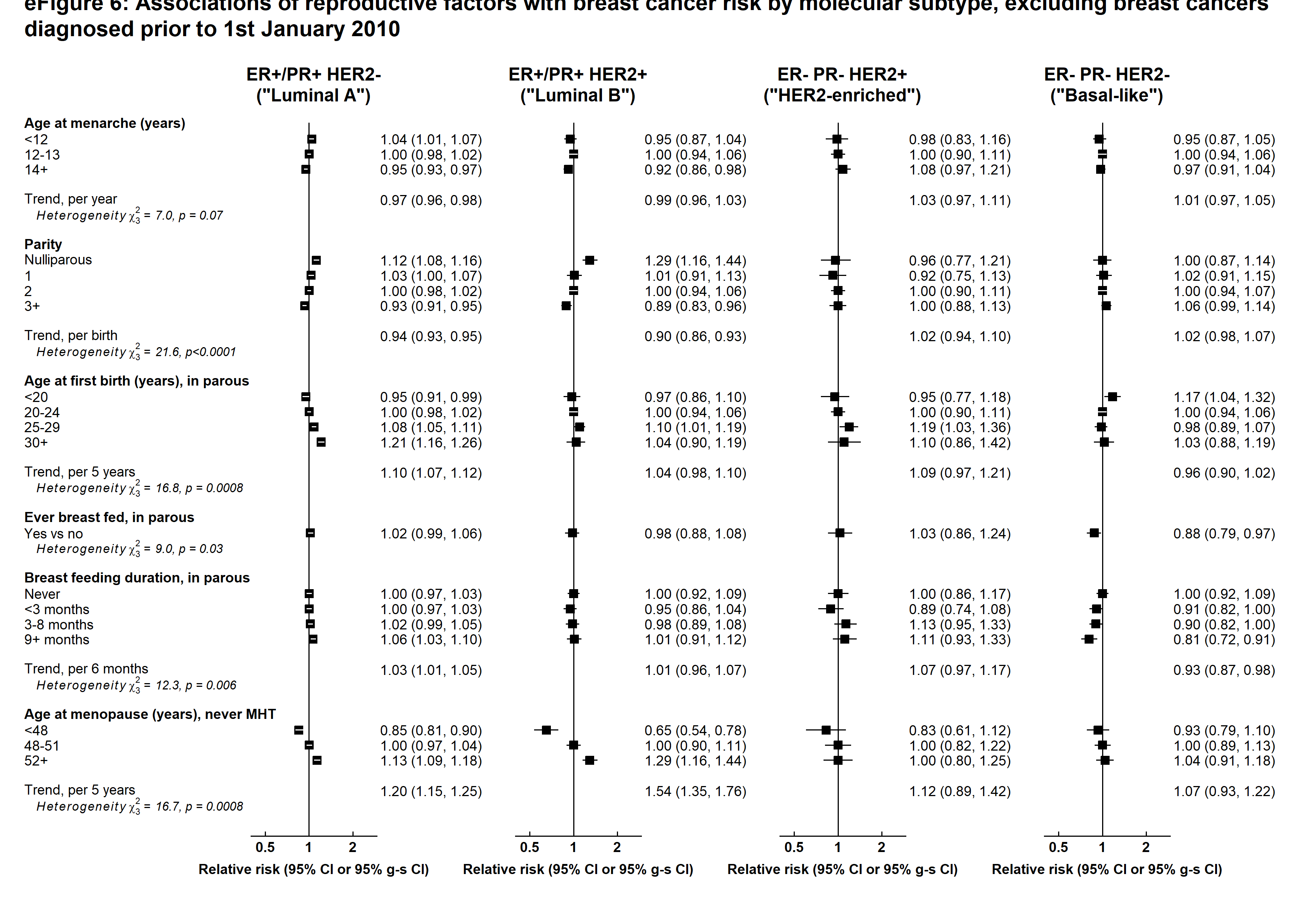


*Tests for heterogeneity are by cancer subtype. g-s CI = group-specific confidence interval*

**eFigure 9: Associations of non-reproductive factors with breast cancer risk by surrogate molecular subtype, excluding breast cancers diagnosed prior to 1^st^ January 2010**


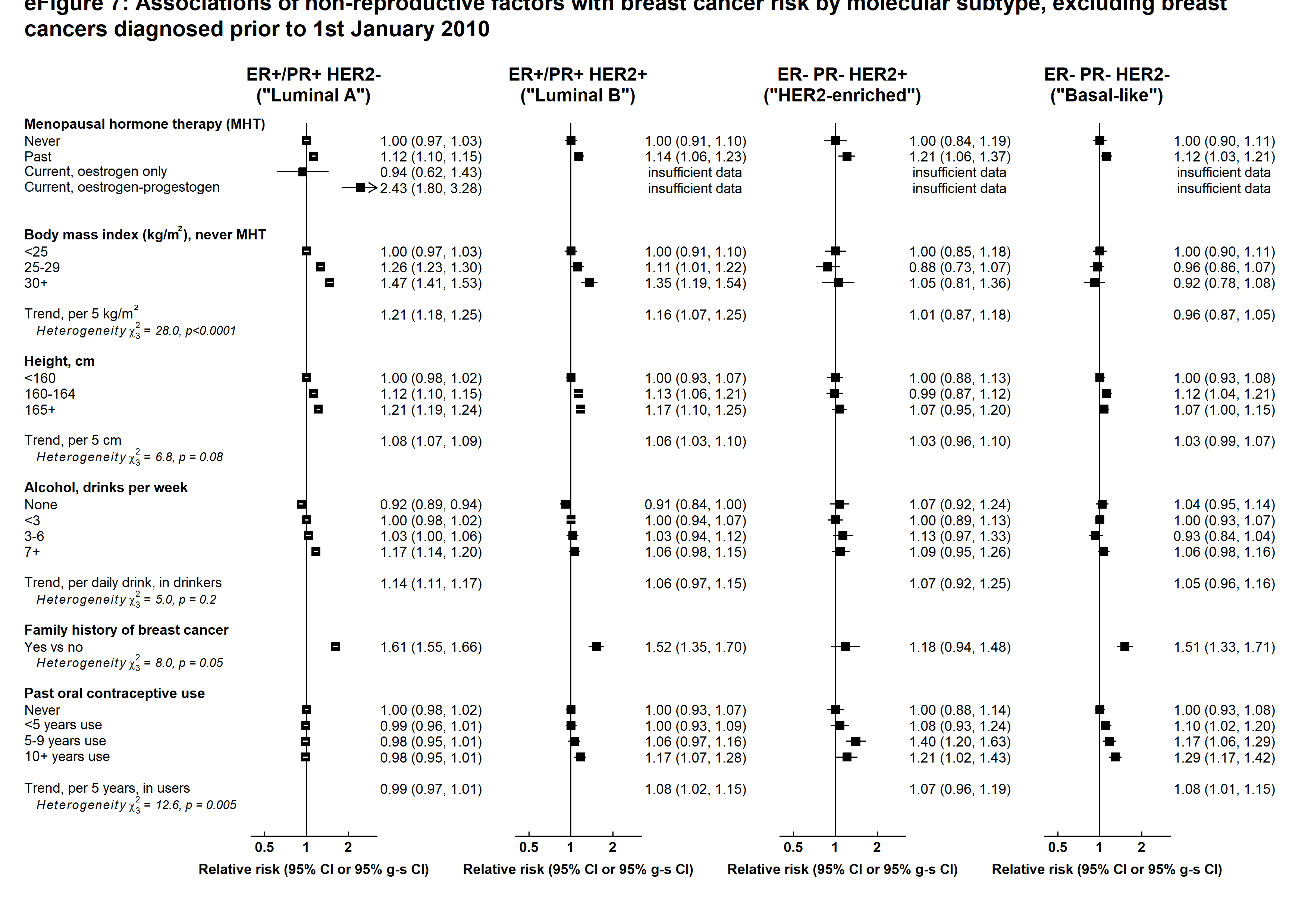


*Tests for heterogeneity are by cancer subtype. Results for groups with fewer than five events are marked as having “insufficient data”. g-s CI = group-specific confidence interval*
